# Mammary Hypertrophy in a Boy

**Published:** 1893-05

**Authors:** 


MAMMARY HYPERTROPHY IN A BOY.—The *Wiener klinische Wochenschrift* for March 16th contains the report of a meeting of the Imperio-royal Society of Physicians of Vienna at which Dr. von Eiselsberg related the case of a boy, fourteen years old, in whom hypertrophy of the right breast had begun about a year before. The progress of the overgrowth had been rather rapid at first, and the size of the breast had since remained stationary. A curious circumstance was that the boy's brother, a few years older, had been affected with a swelling of the left breast, most pronounced about the nipple, shortly after the inception of the first boy's hypertrophy, but it had subsided in the course of a few months.—*Ex.*

